# Implications of irregular shelterwood system on regeneration and species diversity of Sal (*Shorea robusta* Gaertn. f.) forest in Nepal

**DOI:** 10.1016/j.heliyon.2023.e23156

**Published:** 2023-12-02

**Authors:** Niraj Pokhrel, Sachin Timilsina, Nripesh Awasthi, Anita Adhikari, Bikash Adhikari, Santosh Ayer, Kishor Prasad Bhatta

**Affiliations:** a Tribhuvan University, Institute of Forestry, Pokhara Campus, Pokhara, 33700, Nepal; b Ministry of Industry, Tourism, Forest and Environment, Kailali, Sudurpaschim, Nepal; c Tribhuvan University, Institute of Forestry, Hetauda Campus, Makwanpur, 44107, Nepal; d Agriculture and Forestry University, College of Natural Resource Management (CNRM), Katari, 56310, Nepal; e Research and Development Centre, Kathmandu, 44600, Nepal

**Keywords:** Irregular shelterwood system, Seed origin, Species dominance, Timber production

## Abstract

Silvicultural systems are essential for effective forest management and utilize the resources by conserving biodiversity, health, and valuable services forests offer to society. However, Nepal faces a significant knowledge gap due to the limited data on the effect of silvicultural systems and forest management practices on tree diversity and regeneration. Therefore, this study was conducted to assess the effects of canopy opening on natural regeneration and species diversity of Sal (*Shorea robusta* Gaertn. f.) forest in comparison to managed and control blocks in the southern plains of Nepal under irregular shelterwood systems. The vegetation sampling was carried out by the quadrat method (plot of 10 m* 10 m) for studying 48 plots in both managed and control forest areas. The Shannon-Wiener Index and the Simpson Index were used to measure species diversity, whereas Margalef's index was used to measure species richness. Dominant tree species in both managed and control forest areas were identified through the Important Value Index (IVI). The seedling and sapling density of Sal was found to be significantly higher (p < 0.05) in managed blocks. Seedlings of Sal from the seed origin were notably more abundant in the managed block (p < 0.01), whereas those from coppice origin exhibited higher numbers in the control block (p < 0.01). Sal was found to be dominant species in both managed block (IVI = 199.94) and control (IVI = 108.34). The species diversity and richness were significantly declining in the managed forest (p < 0.01) with higher species dominance in comparison to the control blocks. Our study found a positive correlation of canopy cover with species diversity (r = 0.69, p < 0.01) and richness (r = 0.59, p < 0.01) whereas a negative correlation with seedling (r = −0.43, p < 0.01) and sapling density (r = −0.16, p < 0.01). The application of the irregular shelterwood system in study area has effectively promoted natural Sal regeneration while concurrently reducing species diversity. To strike a balance between timber production and biodiversity conservation in these forests, further research focusing on moderating felling intensity within the irregular shelterwood system is strongly recommended.

## 1 Introduction

Forest ecosystems provide an array of goods and services to society for millennia [1]. However, the ever-increasing global population has led to a significant depletion of forest resources, prompting concerns about environmental sustainability [2]. While these concerns and the need for forest management practices are global in scope, it is particularly pertinent to focus on Nepal, a country blessed with rich biodiversity and extensive forest cover [3]. One of the critical elements of forest management in Nepal revolves around the *Shorea robusta* Gaertn. f., commonly known as Sal forests [[4], [5], [6]]. Sal forests in Nepal represent a critical ecological and economic resource, deeply intertwined with the country's identity and well-being [[4], [5], [6], [7]]. These forests, primarily located in the Terai region, have historically played a multifaceted role, contributing significantly to biodiversity conservation, timber production, and the livelihoods of local communities [8,9]. Their immense ecological significance extends beyond the nation's borders, as they are part of the broader Terai-Duar savanna and grasslands ecoregion, a biodiversity hotspot that harbors diverse flora and fauna [10]. However, the sustainability of Sal forests faces multiple challenges such as significantly aged forest exhibiting hollowness, diseased, over-maturity, and poor health due to historical protective management practices which is further exacerbated by increasing human population and demands for timber and non-timber forest products [11].

In response to these challenges, various forest management practices have been adopted, with the irregular shelterwood system under Scientific Forest Management (SciFM) emerging as one notable approach in Terai region of Nepal [[12], [13], [14]]. This system involves the selective removal of mature trees to create canopy gaps, stimulating natural regeneration, while sparing a few “mother” or “shelter” trees and advanced poles for future harvesting [15]. Its significance lies in its potential to balance the extraction of valuable timber resources with ecological sustainability, addressing two critical aspects essential for the well-being of Sal forests—regeneration and tree diversity.

In the lowland Terai, where there are extensive Sal forests, the Government of Nepal (GoN) has also started using the SciFM approach [[16], [17], [18]] to improve the productivity of forests; contribute to the well-being of people and the development of the national economic through a significant quantity of timber production and uses on a commercial basis [19,20]. On the large contiguous block of the Terai region, collaborative forest management has been used to enhance sustainable forest management while taking stakeholders' political, social, and environmental concerns into consideration [21]. It included “closer” (user residing nearby forest) and “distance user” (user residing far from the forest) groups with providing livelihood options, integrating local and traditional forest management practices, and effective benefit-sharing mechanisms between government and communities [22]. Although it has more than 44 % of its area covered in forest, Nepal continued to purchase timber from India and other nearby countries despite these efforts [23]. In Nepal, where 0.83 million cubic meters of timber are imported each year at a cost of USD 52 million, the demand for timber has grown dramatically [[23], [24], [25]]. Therefore, in June 2019, GoN announced discontinuing the SciFM approach because of excessive logging of highly valued timber species including Sal resulting in declining plant diversity and regeneration status [26].

Existence of Sal forests rely on the regeneration process, where young trees replace older ones, to ensure the continued existence of Sal trees and other essential species. For this species, natural regeneration is the only relevant method of regeneration [27] and silvicultural operations are required to improve the productivity [28]. However, the success of this regeneration process is intrinsically linked to various factors such as rainfall, seed year, light availability etc. [29]. For instance, Sal seeds exhibit an impressive germination rate, exceeding 90%, but depend on timely rainfall within a week. Seeds of Sal remains viable for just about a week, so monsoon delays in late June can hinder successful germination, risking seedling failure [29]. Another important factor to consider is the seed production by Sal trees. Sal trees consistently produce seeds every year, with a particularly abundant seed production occurring typically every third year [29]. Similarly, Sal trees are categorized as light-demanding species, needing ample overhead light throughout their development, even from germination [30]. The forest canopy plays a pivotal role in either facilitating or hindering regeneration, directly influencing the growth of understorey seedlings and saplings [31]. Therefore, light availability is a fundamental factor in the growth and sustainability of Sal forests. Sal forests are not monolithic entities; they are rich and diverse ecosystems, harboring a wide variety of tree species alongside the dominant Sal [32]. This diversity contributes to the resilience of the forest in several ways. Firstly, it helps protect against diseases and pests, as different tree species have varying vulnerabilities [33]. Secondly, it enhances the habitat for a diverse range of wildlife species, providing them with food sources, nesting sites, and shelter [34]. Furthermore, diverse tree populations offer a broader range of ecosystem services, including carbon sequestration, soil stabilization, water purification, and the provision of valuable non-timber forest products [35]. Therefore, understanding the intricate dynamics of Sal regeneration and recognizing the vital role of tree diversity is fundamental to comprehending the implications of the irregular shelterwood system on these forests' regeneration and species diversity [12,36].

The research gap pertaining to the implications of the irregular shelterwood system on Sal forest regeneration and tree diversity in Nepal represents a critical void that demands immediate attention and exploration. Prior studies have made commendable strides in understanding various facets of Sal forest ecosystems, including aspects of structure, composition, species diversity, and regeneration dynamics [12,15,[37], [38], [39], [40], [41]]. However the specific implications of the irregular shelterwood system on Sal forest regeneration and tree diversity remain understudied. This gap needs to be filled for several compelling reasons. Firstly, the irregular shelterwood system holds the promise of sustainable Sal forest management, and uncovering its precise impacts on regeneration and tree diversity is pivotal for informed, ecologically sound forest management strategies [42]. Secondly, Sal forests are vital components of Nepal's ecosystems, supporting diverse flora and fauna [5,6], and studying the system's effects on regeneration and diversity is crucial for their long-term ecological health and resilience. Moreover, these forests are integral to the livelihoods of local communities [8,9], emphasizing the need for insights that can enhance community well-being through sustainable resource utilization. Addressing this research gap is not only timely but also essential for the conservation and effective management of Sal forests in Nepal especially in light of the ban on SciFM in Nepal due to concerns of excessive logging and its impact on declining plant diversity and regeneration status [43], offering valuable lessons for the broader discourse on forest sustainability and management practices globally. Therefore, this study aimed to assess the effect of canopy opening due to an irregular shelterwood system on species diversity and regeneration potential of one of the important hardwood species (i.e., *S. robusta*) of Nepal. The findings from this study will have direct implications for policy and management decisions, facilitating the balance between timber extraction and ecological sustainability.

## 2 Methodology

### 2.1 Description of the study sites

**Fig. 1:**
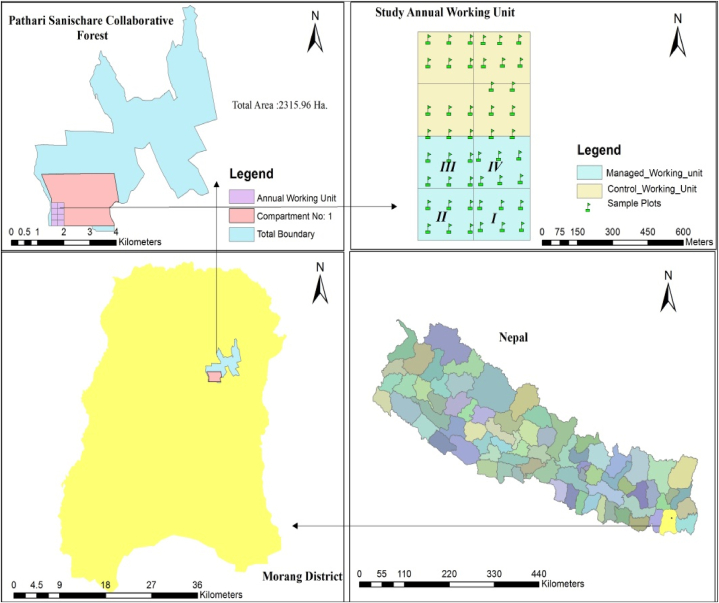
Map of study area.

The study was carried out in the Pathari Sanischare Collaborative Forest, which is situated in Pathari-Sanischare, Urlabari, Letang Municipality and Miklanjung Rural Municipality, of Morang District of Eastern Terai region of Nepal (Fig. 1). Pathari Sanischare Collaborative Forest site covers an area of 2315.96 ha. Out of the total forest area, 1913.17 ha is productive forest, located between 26⁰ 40′50.63″ N to 26⁰ 45′0.81″ N latitudes and 87⁰ 31′57.11″ E to 87⁰ 36′9.95″ E longitudes. The elevation ranges from 85 m to 185 m from the mean sea level (msl). This collaborative forest includes a 2, 27,715 population from a total of 51,333 user households, among them 1,07,177 are male and 1,19,908 are female. The average annual temperature and precipitation of the district are 25.2 °C and 1870 mm per year respectively.

**Fig. 2:**
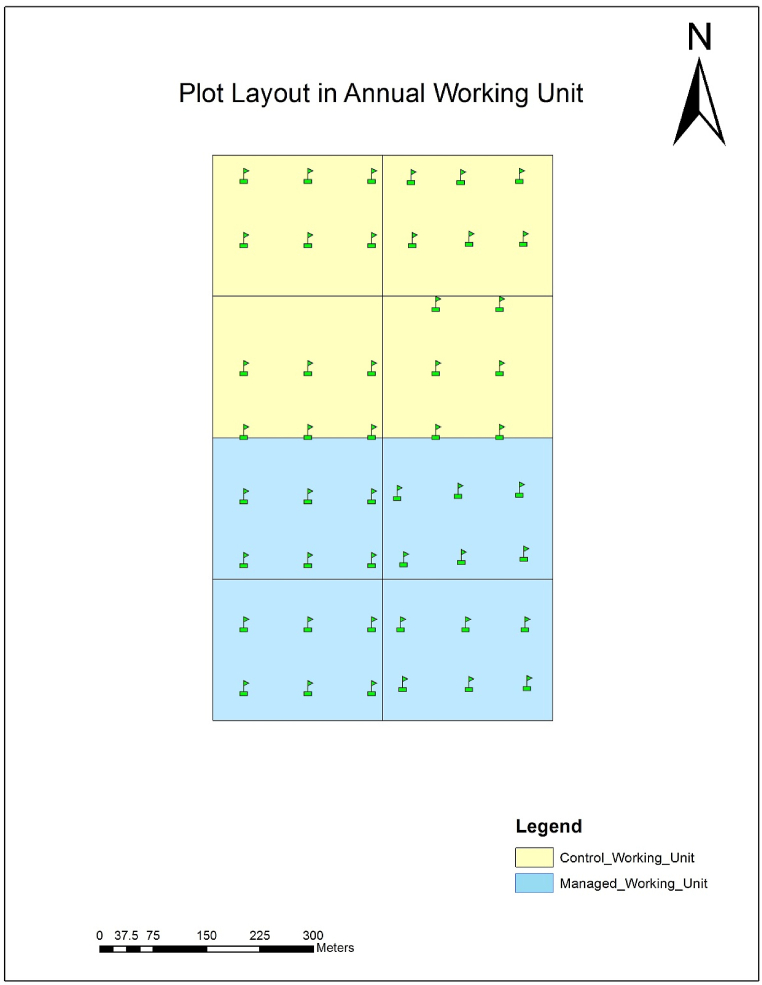
Plot layout in working unit.

The irregular shelterwood system was employed in the study area in 2016. The total forest area is divided into five compartments and each compartment is into 8 sub-compartment finally sub-compartments are into 10 annual working units. Compartment 1 and compartment 2 are Sal dominated whereas the rests of the others are dominated by low economically valuable species like *Mallotus philippinensis, Dillenia pentagyna, Stereospermum chelonoides.* Along with the regeneration felling weeding, cleaning was done every year in each regeneration felling area and thinning in the first annual working unit in the year 2020 (Table 1). The general stand characteristics such as management age, basal area. tree density, canopy cover, felling intensity and retention of mother trees of both managed and control blocks are depicted in Table 2. The study was carried out in C1s1 (Compartment 1 sub-compartment 1) which is Sal dominated forest covering an area of 47.57 ha (Fig. 2). Each annual working unit is an equi-extensive area of 4.74 ha. The four-regeneration felling area of a total of 18.96 ha had been harvested following an irregular shelterwood system. In this regeneration felling area, total of 246 mother trees was left for natural regeneration as well as for the prevention of soil erosion.

**Table 1:** Block-wise management intervention in each fiscal year.

Blocks/Fiscal year	2016/2017	2017/2018	2018/19	2019/2020
**Block I (4 years old)**	Regeneration Felling	Removal of lops and tops; cleaning, and removal of unwanted or undesirable/climber species;	Removal of lops and tops, cleaning, removal of unwanted or undesirable/climber species;	Removal of lops and tops; cleaning: removal of unwanted or undesirable/climber species; Thinning
**Block II**		Regeneration Felling	Removal of lops and tops; cleaning: removal of unwanted or undesirable/climber species;	Removal of lops and tops; cleaning: removal of unwanted or undesirable/climber species;
**Block III**			Regeneration Felling	Removal of lops and tops; cleaning: removal of unwanted or undesirable/climber species;
**Block IV (1 year old)**				Regeneration Felling
**Control Unit**	No intervention

**Table 2:** General *stand characteristics of the study sites*.

Blocks	Age (years of management)	Basal area (m^2^ha^−1^)	Tree density ha^−1^ (Diameter at breast height-dbh > 10 cm)	Canopy cover (%)	Felling intensity (trees ha^−1^)	mother tree (trees ha^−1^)
**Managed blocks**	Average (1–4 years stand)	25.80	500	40.80	45.09	12.97
**I**	4-year	21.83	300	36.37	16.24	15.18
**II**	3-year	26.00	683	45.38	62.86	14.34
**III**	2-year	26.33	716	45.16	53.58	11.39
**IV**	1-year	28.83	300	36.23	47.67	10.97
**Control**	No	77.20	570	63.70	No	–

### 2.2 Data collection

**Fig. 3:**
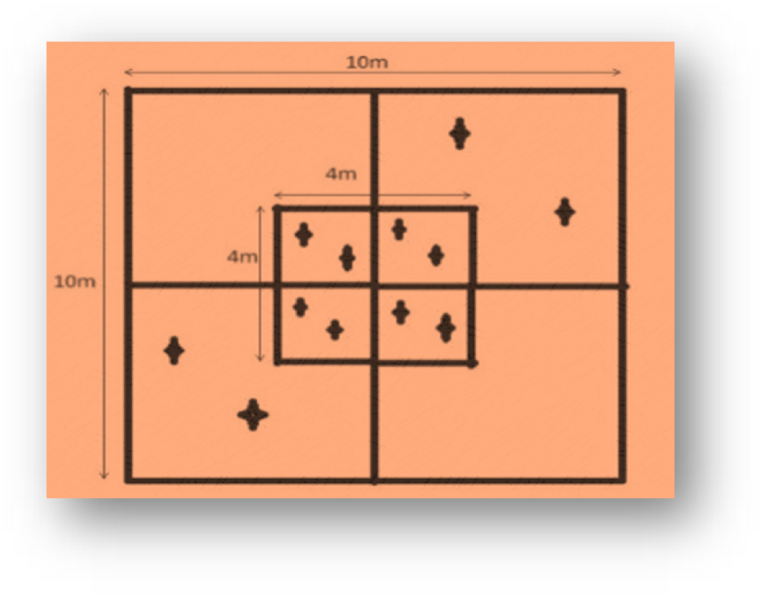
Plot design for recording forest variables.

The Quadrat method [44] was adopted for vegetation sampling in the study area (Fig. 3). The quadrant of size 10 m*10 m for trees (dbh>10 cm) was laid out based on the principle of simple random sampling with the help of GIS, while two opposite quadrants of 5 m*5 m were for measuring seedlings (height-ht<1.3 m) and saplings (dbh<10 cm and ht > 1.3 m), as determined by Species Area Curve Method [44]. Similarly, the nested sub-quadrat of 4 m*4 m was laid out for taking data on the (mode) origin of *S. robusta* (seedlings either from seed or root sucker or coppice) with the help of a soil digger. The origin of *S*. *robusta* seed and seedling coppice is considered as seed origin and rest of the others are as coppice origin. The sampling intensity ≥1 % based on community forest inventory guideline was used [45]. Altogether 48 plots, 6 plots for each managed and control block (i.e., 6 plots *4 blocks = 24 for managed blocks and 24 for control) were laid to study the different forest variables. Within plots, the total height and diameter at breast height (dbh) of trees and saplings were measured using a Range finder and diameter tape along with the height of seedlings by diameter tape. The canopy cover of the plots was measured by a spherical densiometer from the four corners and the center of the plots and an average value was accounted for each plot.

### 2.3 Data analysis

Firstly, the Shapiro-Wilk test was used to assess the normality of the collected data which showed the data were not normally distributed [46]. The stand characteristics such as basal area, tree density, frequency and canopy cover of both managed and control blocks were calculated [47] and analyzed using descriptive statistics. The density of seedling/saplings was computed, and block-wise comparisons were tested by one way ANOVA where management regime-based comparisons were made by t-independent and post hoc Tukey test in R-studio [48]. Similarly, the Importance Value Index (IVI) was assessed for each species to get information on the dominant species of both managed and control blocks. The relative density, relative dominance, and relative frequency of each species were measured in order to determine the IVI [47].

Moreover, plant diversity was studied using Shannon Wiener's Index [49], Simpson's Dominance Index [50], Margalef's Species Richness Index [51], Equitability or Evenness Index [52], and Jacard's Similarity Index [53]. Pearson's correlation coefficient [54] was performed in R studio [48] for determining the relation between regeneration density and diversity with canopy cover. For the mode of regeneration either from seed or root sucker and or coppice, from the destructive sampling method collar diameter of each sample of Sal seedling was measured and analyzed by using descriptive statistics as in seedlings from seed, where the root of seedlings vertically go downwards and absent of auxiliary horizontal root, in root sucker origin shoot above ground and below ground diameter has almost similar and in coppice originated seedling diameter of shoot above ground has smaller that of root diameter.

## 3 Results

### 3.1 Regeneration status

The mean seedling and sapling density was found comparatively higher (p < 0.05) in the managed blocks than in control block (Table 3). Similarly, the seedling and sapling density of Sal was also significantly higher in managed blocks. Regarding the origin of seedlings, the percent of seed origin in Sal seedlings was found significantly higher (p < 0.05) in the managed block while coppice origin seedlings were higher in the control blocks (Table 3).

**Table 3:** Mean regeneration attributes of managed and control blocks.

Parameters	Managed blocks	Control blocks	p-value
**Seedling density (ha**^**−**^**^1^)**	10,425	6033	<0.001
**Sapling density (ha**^**−**^**^1^)**	3500	2700	0.011
**Seedling density (Sal, ha**^**−**^**^1^)**	9391	3241	<0.001
**Sapling density (Sal, ha**^**−**^**^1^)**	2958	1233	<0.001
**Seedling Percent (seed origin Sal)**	58.77	48.52	<0.001
**Seedling Percent (coppice origin Sal)**	41.22	51.47	<0.001

**Fig. 4:**
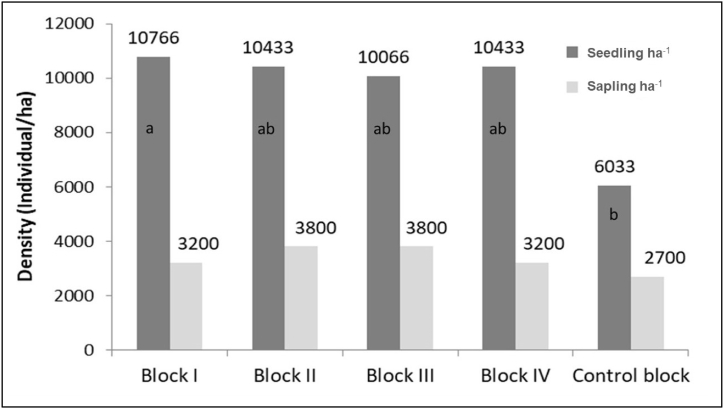
Seedling and sapling density of different blocks. Different letters indicate significant differences between means according to post hoc Tukey' HSD test (p < 0.05).

Specific to the studied blocks, seedling density was found highest (10,766 ind ha^−1^) in managed block I while the lowest (6033 ind ha^−1^) in control block (Fig. 4). The remaining managed blocks (II, III, and IV) had almost similar seedling densities (>10,000 ind ha^−1^). The ANOVA revealed a statistically significant difference in seedling density among different blocks (p < 0.05). Further, post hoc Tukey test revealed that control block had significantly lower seedling density compared to Block I (p < 0.05), but there were no significant differences among the other blocks or between pairs of those blocks (p > 0.05). However, there was no statistically significant difference (p > 0.05) in sapling density among the different blocks (Fig. 4).

**Fig. 5:**
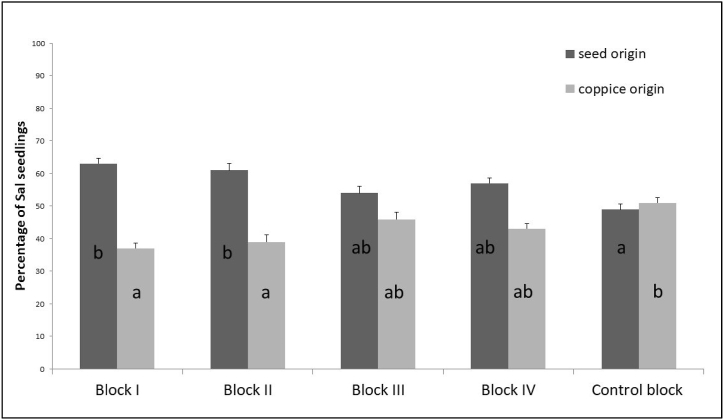
Regeneration origin of Sal. Different letters indicate significant differences between means according to post hoc Tukey' HSD test (p < 0.05).

Regarding the regeneration origin, ANOVA test revealed that there was statistically significant difference (p < 0.01) in seedling density of seed origin among the different blocks (Fig. 5). The post hoc Tukey test revealed that the seedling density of Sal of seed origin in the Control block was significantly lower than in Block I and II (p < 0.05) while no significant differences in seedling density of seed origin were observed among the other blocks (p > 0.05). Similarly, there was statistically significant difference (p < 0.01) in seedling density of coppice origin among the different blocks. The post hoc Tukey test revealed that the seedling density of coppice origin in the Control block was significantly higher than in Block I and II (p < 0.05) and no significant differences in seedling density of coppice origin among other blocks (p > 0.05) (Fig. 5).

The mean collar diameter and height of seed origin seedling of Sal are 1.00 cm and 46.08 cm respectively in a managed block, while 0.45 cm and 30.10 cm respectively in the control block. Similarly, the mean collar diameter and height of coppice origin seedling of Sal are 1.74 cm and 86.58 cm respectively in a managed block which is 0.87 cm and 68.49 cm respectively in control blocks.

**Fig. 6:**
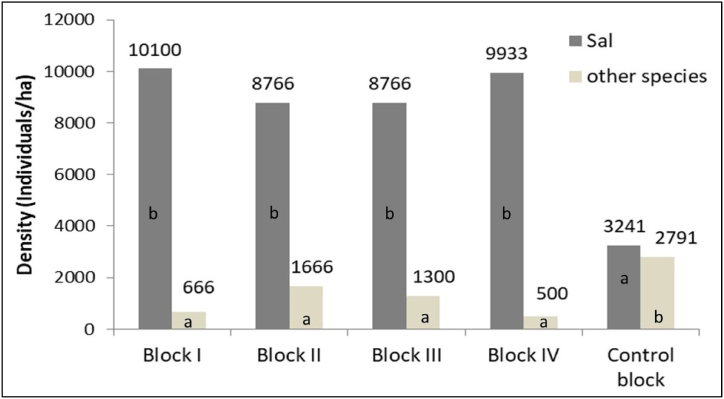
Seedling density of Sal and other species. Different letters indicate significant differences between means according to post hoc Tukey' HSD test (p < 0.05).

**Fig. 7:**
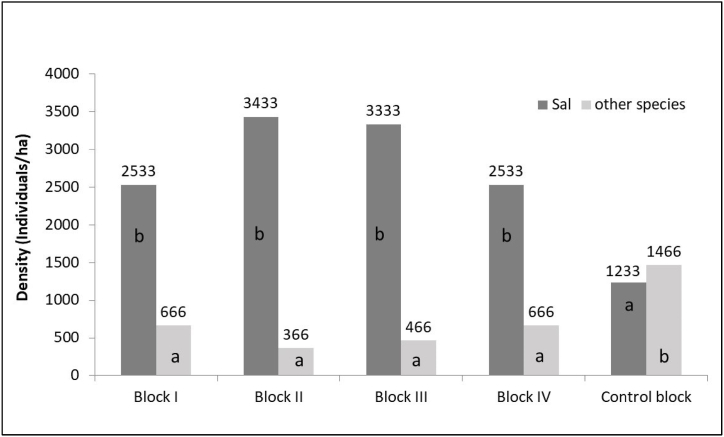
Sapling density of Sal and other species. Different letters indicate significant differences between means according to post hoc Tukey' HSD test (p < 0.05).

The huge contrast between regenerations of Sal and other species was observed with remarkable dominance of Sal seedlings and saplings in managed blocks with regards to other species (Fig. 6, Fig. 7). The ANOVA tests revealed that there was statistically significant difference (p < 0.01) in seedling density of both Sal and other species. In the case of Sal seedlings, the post hoc Tukey test showed that the seedling density in the Control block was significantly lower than in Block I, II, III, and IV (p < 0.05). Conversely, for other species, the post hoc Tukey test revealed that the seedling density in the Control block was significantly higher than in Block I, II, III, and IV (p < 0.05). However, there were no significant differences (p > 0.05) in seedling density observed among Blocks I, II, III, and IV for either Sal or other species (Fig. 6).

Similarly, ANOVA test also revealed that there was statistically significant difference (p < 0.01) in sapling density among different blocks for both Sal and other species (Fig. 7). For Sal saplings, a post hoc Tukey test showed that the Control block had significantly lower sapling density compared to Blocks I, II, III, and IV (p < 0.05). Conversely, for other species, the post hoc Tukey test revealed that the Control block had significantly higher sapling density compared to Blocks I, II, III, and IV (p < 0.05). However, there are no significant differences between Block I, II, III and IV in sapling density for both Sal and other species (Fig. 7).

### 3.2 Tree community structure and compositions

**Fig. 8:**
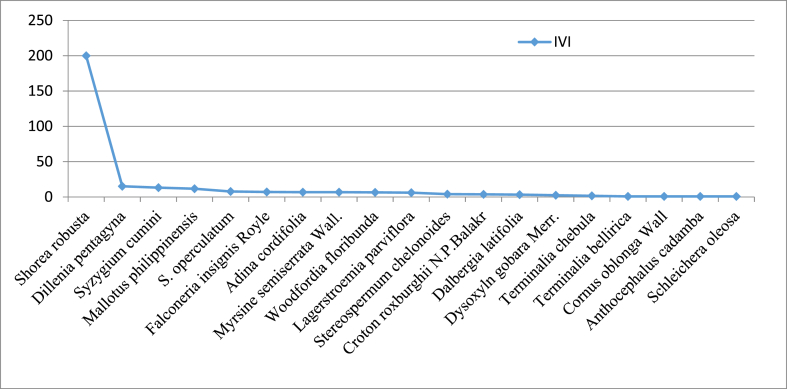
Dominance diversity curve of the managed block.

**Fig. 9:**
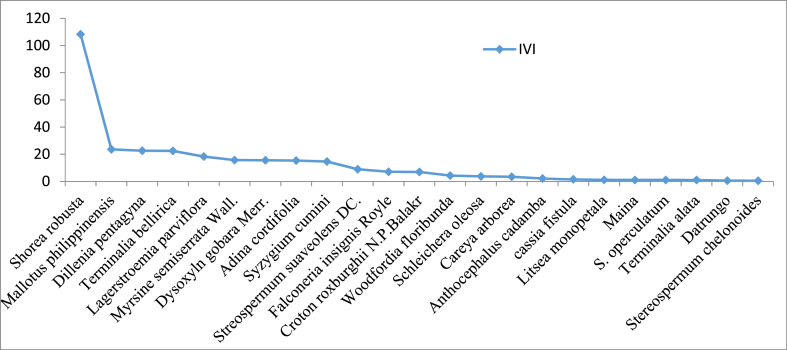
Dominance diversity curve of unmanaged block.

The number of tree species recorded in managed and control blocks was 19 and 23 respectively. Among them, Sal found to be the dominant species in both managed and control with IVI values of 199.94 and 108.34 respectively (Fig. 8, Fig. 9). Similarly, *Dillenia pentagyna* (with IVI value, 15.21) and *Mallatus phillipensis* (with IVI value, 23.57) was found as co-dominant species in managed and control block respectively. The details of the species recorded in both managed and control blocks with their IVI values are provided in Appendix I.

### 3.3 Species diversity and richness

The mean value of the Shannon diversity index (H) was significantly different between the managed and control blocks with higher diversity in the control block (1.63 ± 0.04) and lowest diversity in managed block IV (0.43 ± 0.12) (Table 4). However, the concentration of dominance (C) was found inversed to that of Shannon's diversity, higher in managed blocks (0.79 ± 0.02) than in control block (0.30 ± 0.01). The species evenness (E) and richness index (S) was significantly higher in control blocks than in managed one (Table 4). The species richness was lowest in block III (0.89 ± 0.13), among the managed blocks.

**Table 4:** Species diversity indices of different studied blocks.

Blocks	H	C	E	S
**I**	0.47 ± 0.25	0.81 ± 0.05	0.26 ± 0.05	1.04 ± 0.25
**II**	0.66 ± 0.09	0.72 ± 0.03	0.34 ± 0.03	1.36 ± 0.16
**III**	0.51 ± 0.07	0.77 ± 0.03	0.32 ± 0.02	0.89 ± 0.13
**IV**	0.43 ± 0.12	0.82 ± 0.04	0.25 ± 0.05	0.97 ± 0.24
**Average of the managed block**	0.52 ± 0.05	0.79 ± 0.02	0.30 ± 0.02	1.06 ± 0.10
**Control**	1.63 ± 0.04	0.30 ± 0.01	0.74 ± 0.02	2.21 ± 0.10
**p-value**	<0.01	<0.01	<0.01	<0.01

Where: H: Shannon-diversity; C: Concentration of dominance; E: Evenness/Equitability index; S: Species richness index.

To determine resemblance, the Jacard Similarity Index was used. The greatest similarity was found between Block I and Block IV of managed forest which means 80 % of species are common to both blocks (Table 5). Meanwhile, the similarity of species between managed and a control block is just greater than 50 %. Ground operation (control burning) and tending operations like weeding and cleaning are the main cause of the reduction in similarity. On average among recorded species, 62 % are found both in managed and control blocks (Table 5).

**Table 5:** Jacard's similarity index between studied blocks.

Site/practices	Similarity value
Block I versus Block II	72
Block I versus Block III	69
Block I versus Block IV	80
Block I versus Control Block	54
Block II versus Block III	76
Block II versus Block IV	67
Block II versus Control block	54
Block III versus Block IV	63
Block III versus Control block	52
Block IV versus Control block	50

### 3.4 Relation of regeneration and species diversity with canopy cover

The seedling and sapling density was negatively correlated with canopy cover and the overall seedling and sapling density of Sal was found statistically significant with correlation coefficient values of −0.43, −0.54, and −0.49 respectively (Table 6). Similarly, the seed origin seedling was also found significantly negatively correlated with canopy cover whereas coppice origin seedlings were found positively correlated with canopy cover (Table 6).

**Table 6:** Correlation between canopy cover and regeneration attributes.

Pearson correlation (r)	Seedling density	Sapling density	Sal seedling density	Sal sapling density	Seed origin Sal seeding	Coppice origin Sal seedlings
**Canopy cover**	−0.43^a^	−0.16	−0.54^a^	−0.49^a^	−0.43^a^	0.43^a^

*Correlation is significant at the 0.05 level (2-tailed).

a Correlation is significant at the 0.01 level (2-tailed).

The Shannon's diversity, evenness index, and species richness index were found significantly positively correlated (p < 0.01) with canopy cover while it was negative for the concentration of dominance (Table 7).

**Table 7:** Correlation coefficient (r) between canopy cover and diversity indices.

Pearson correlation (r)	H	C	E	S
**Canopy cover**	0.69^a^	−0.69^a^	0.66^a^	0.59^a^

*Correlation is significant at the 0.05 level (2-tailed).

a Correlation is significant at the 0.01 level (2-tailed).

## 4 Discussion

Sal, commonly known as Sal, is a light-demanding species crucially reliant on overhead solar light for its growth and establishment, particularly during the critical regeneration stages [12,55]. We observed a noteworthy difference in the density of Sal seedlings and saplings between managed blocks and control blocks (Fig. 4). Our findings align with Awasthi et al. [11] and Belbase et al. [56], that underscores the critical role of light availability in Sal regeneration. They both observed a substantial increase in regeneration when canopy openings, resulting from regeneration felling's, enhanced light availability in Sal forest of central and western Nepal. The observed success of the irregular shelterwood system in our study further underscores the importance of light availability for the regeneration of this light-demanding species. Additionally, our study aligns with the broader discourse on forest regeneration, as emphasized by Gautam et al. [57], Neupane et al. [58] and Bhatta et al. [59], who highlighted the importance of controlling disturbance intensity to support regeneration. The controlled disturbances created by this silvicultural system appear to have contributed to the enhanced regeneration of Sal in the managed blocks. Therefore, our study not only reaffirms the critical role of light availability and the success of the irregular shelterwood system in Sal regeneration but also aligns with the broader consensus on the need for controlled disturbances in sustaining healthy forest ecosystems.

The distribution of Sal seedlings based on their origin bears significance for understanding forest dynamics, guiding management strategies, and informing conservation efforts. Our study revealed a higher prevalence of seed-origin Sal seedlings in managed blocks, and coppice origin Sal seedlings in controlled blocks (Fig. 5). The prevalence of seed-origin seedlings in managed blocks underscores the pivotal role of controlled canopy management, a characteristic of the irregular shelterwood system applied in these areas. This system creates canopy openings that allow increased sunlight penetration - a critical factor for Sal, a light-demanding species [60]. The availability of ample sunlight and reduced competition for resources, including nutrients and moisture in managed blocks fosters favorable conditions for the regeneration of seed-origin seedlings. Conversely, control blocks, with their undisturbed nature and limited canopy openings, exhibited a higher proportion of coppice-origin seedlings. This observation highlights the species' remarkable capacity to regenerate through coppicing even in less favorable or adverse conditions. Coppice-origin seedlings, originating from rootstocks, have the advantage of efficiently utilizing available resources, including nutrients and moisture. They are also well-equipped to withstand the challenges posed by competition in the absence of active management practices [60]. The findings from our study align with previous research by Suoheimo [61] in Sal-dominated forests, who reported a similar pattern of predominantly coppice-origin seedlings in natural settings. Both coppice and seed-origin trees produce fertile seeds, and the vigor of resulting seedlings is similar [31]. However, seed-origin seedlings contribute to genetic diversity, enhancing adaptability to changing environments over time [29,62]. This insight underscores the importance of management practices, such as the irregular shelterwood system, which can create favorable conditions for seed-origin seedlings. By carefully controlling canopy openness and environmental factors, this system can not only support the immediate regeneration of Sal but also contribute to the long-term vitality and adaptability of these forests.

We observed that Sal was the dominant species in both the managed and control blocks (Fig. 8, Fig. 9). This finding aligns with previous studies [12,36,63,64] in Sal forest under irregular shelterwood system in Nepal. This dominance of Sal can be attributed to a combination of ecological, economic, and management factors. One of the reasons for Sal prominence in the studied forest may be the active protection and recognition of its ecological significance and essential ecosystem services by the local community [65]. While active management efforts in the managed block may contribute to its prominence, passive protection and historical practices may have played a role in the control block. Sal is renowned for its valuable timber, which serves as a primary resource for construction, furniture making, and fuelwood. Additionally, it provides non-timber forest products such as resin, leaves for plates and thatching, and medicinal substances, which have local and commercial value. Such recognition of the multifaceted benefits of Sal may lead to conservation efforts aimed at preserving its dominant status within the forest ecosystem. Furthermore, the irregular shelterwood system also plays a key role in maintaining dominancy of Sal in Nepalese context. This system involves the selective removal of trees to create canopy gaps for promoting growth of other retained species, and it is a common practice to prioritize economically valuable species, notably Sal, for retention. This selective removal of other less valued tree species minimizes competition for vital resources, including sunlight and nutrients, and creates favorable conditions for the growth and sustainability of economically significant species like Sal. Our study has also shed light on the presence of opportunistic species within the forest ecosystem benefiting from canopy openings, with *Mallotus philippensis* and *Dillenia pentagyna* serving as a notable example (Fig. 9). The presence of opportunistic species should be factored into forest management plans, especially those involving canopy disturbances. Understanding how opportunistic species respond to forest management practices can guide decisions on selective tree removal, habitat conservation, and sustainable timber harvesting [64].

There is low species diversity, richness, and evenness in the managed blocks compared to the control blocks, highlighting the significant impact of an irregular silvicultural system (Table 4). These findings are consistent with prior research [12,36,63] in Sal forest under irregular shelterwood system in Nepal. This can be attributed to practices such as harvesting, logging, and the removal of unwanted vegetation, and retention of high valued species such as Sal which is common in managed blocks [36]. In contrast, Smith et al. [66] found that in irregular shelterwood-managed stands, species diversity initially decreases after logging due to disturbance but then gradually recovers and increases as the forest matures, offering diverse habitats. Therefore, this system might have the potential to increase diversity in the long run, particularly as the forest ecosystem recovers and matures. However, this hypothesis needs to be tested with further research. Conversely, the control block exhibited higher species diversity, evenness, and richness, underscoring the ecological value of areas with minimal or no active management. Such areas can function as sanctuaries for a diverse array of species and contribute to the overall resilience of the ecosystem [67]. Therefore, managing forests presents a complex challenge that necessitates a delicate balance between economic objectives, such as timber harvesting, and ecological objectives, including biodiversity conservation. Addressing these complexities requires a careful consideration of the long-term ecological consequences and the implementation of adaptive management strategies aimed at fostering both economic and ecological resilience.

Our study found a negative correlation between seedling and sapling density of Sal and canopy cover (Table 6). This finding aligns with previous studies [12,36,63] where they reported higher seedling and sapling density of Sal in managed block with canopy opening than control blocks. Similarly, Baral and Ghimire [68] also found highest regeneration density of Sal in 0–25 % canopy cover and lowest in 75–100 % canopy cover. This suggests that as the canopy cover increases, the density of Sal seedlings and saplings decreases. This negative correlation could be attributed to the shading effect of a denser canopy, which limits the amount of sunlight reaching the forest floor [42]. Sal, like many other tree species, require more open conditions to establish and grow successfully [29]. Therefore, it is necessary to maintain appropriate canopy openings for supporting regeneration of Sal. Similarly, seed origin seedlings showed a significant negative correlation (Table 6), indicating that they can thrive better in less shaded conditions. On the other hand, coppice origin seedlings exhibit a positive correlation with canopy cover (Table 6), suggesting they may be more adapted to growing under a denser canopy. This divergence in responses underscores the importance of considering the origin of seedlings when planning forest management strategies. It might be necessary to create suitable microenvironments for both seed origin and coppice origin seedlings to ensure their successful regeneration [29].

Our study found positive correlation of canopy cover with Shannon's diversity, evenness, and species richness and negative correlation with concentration of dominance is a promising result (Table 7). This finding aligns with previous studies [12,36,63] where they found higher species diversity in control blocks with higher canopy cover in comparison to managed blocks with lower canopy cover. This finding also aligns with the general understanding from ecological theories such as Intermediate Disturbance Hypothesis [69], Resource Partitioning [70], Habitat Heterogeneity [71]. These theories states that denser canopies in a forest create varying light conditions and microhabitats on the forest floor [72,73] This heterogeneity allows different plant species to thrive in different parts of the forest, contributing to higher Shannon's diversity, evenness, and species richness [71]. Similarly, the reduced availability of resources like light under a denser canopy can reduce competition among plant species [70]. This reduction in competition allows multiple species to coexist and utilize the available resources more efficiently, further enhancing biodiversity. Also, denser canopies limit the dominance of any one species by restricting the amount of light it receives [18]. This contributes to a more balanced and biodiverse forest ecosystem, as no single species can monopolize resources [74]. Therefore, understanding relation of canopy cover with different factors and how denser canopies contribute to increased biodiversity, reduced dominance, and a more balanced forest ecosystem is crucial for forest management. And a balance between maintaining canopy cover for ecological diversity and creating suitable conditions for the regeneration of valuable tree species like Sal is suggested.

## 5 Conclusion

The silvicultural practices under the irregular shelterwood system are effective to promote and establish the seedling and sapling density of the intended species (Sal). The increasing regenerations of intended species reflect the future productivity and profitability of the forests. Also, leaving the forest unmanaged may lead to increasing species richness and density of other species than Sal. Similarly, Sal seedlings are better established originated from seeds in managed forest than the natural stands.

## Funding

No funding available for this research.

## Data availability statement

Data will be made available on request.

## CRediT authorship contribution statement

**Niraj Pokhrel:** Writing – original draft, Methodology, Investigation, Formal analysis, Data curation. **Sachin Timilsina:** Writing – review & editing, Data curation, Conceptualization. **Nripesh Awasthi:** Supervision, Methodology, Formal analysis, Conceptualization. **Anita Adhikari:** Visualization, Validation, Formal analysis. **Bikash Adhikari:** Writing – review & editing, Supervision. **Santosh Ayer:** Writing – review & editing, Data curation. **Kishor Prasad Bhatta:** Writing – review & editing, Writing – original draft.

## Declaration of competing interest

The authors declare that they have no known competing financial interests or personal relationships that could have appeared to influence the work reported in this paper.

## Figures and Tables

**Table:** 

			IVI of different species				
SN	Species name	Family	block I	block II	block III	block IV	Control
1	*Shorea robusta*	Dipterocarpaceae	209.19	181.53	201.13	211.08	108.30
2	*S. operculatum*	Myrtaceae	10.39	5.55	4.01	11.46	1.06
3	*Dillenia pentagyna*	Dilleniaceae	13.09	24.28	15.82	11.04	22.61
4	*Adina cordifolia*	Rubiaceae	10.29	2.66	4.01	11.22	15.39
5	*Mallotus philippinensis*	Euphorbiaceae	10.60	14.35	9.38	11.46	23.58
6	*Myrsine semiserrata Wall.*	Ebenaceae	9.96	5.77	4.01	7.40	15.70
7	*Dysoxyln gobara Merr.*	Maliaceae	3.24	2.66	3.79	0.00	15.56
8	*Terminalia bellirica*	Combretaceae	3.45	0.00	0.00	0.00	22.44
9	*Falconeria insignis Royle*	Euphorbiaceae	6.71	9.15	4.21	7.39	7.07
10	*Stereospermum chelonoides*	Bignoniaceae	6.50	2.66	0.00	7.16	0.46
11	*Syzygium cumini*	Myrtaceae	6.52	13.56	23.13	7.17	14.61
12	*Lagerstroemia parviflora*	Lythraceae	3.33	8.68	8.50	3.64	18.32
13	*Alangium salviifolium*	Alangiaceae	3.26	9.10	10.41	3.58	4.29
14	*Dalbergia latifolia*	Fabaceae	3.46	5.33	0.00	3.82	0.00
15	*Cornus oblonga Wall*	Cornaceae	–	2.66	0.00	0.00	0.00
16	*Terminalia chebula*	Combretaceae	–	2.67	3.80	0.00	0.00
17	*Croton roxburghii N.P.Balakr*	Euphorbiaceae	–	6.71	7.80	0.00	6.99
18	*Anthocephalus cadamba*	Sapindaceae	–	2.66	–	–	2.12
19	*Schleichera oleosa*	Rubiaceae	–	–		−3.57	3.76
20	*Streospermum suaveolens DC.*	Bignoniaceae	–	–	–	–	8.96
21	*Litsea Monopetela*	Lauraceae	–	–	–	–	1.15
22	*cassia fistula*	Leguminosae	–	–	–	–	1.59
23	*Careya arborea*	Lecythidaceae	–	–	–	–	3.45
24	*Datrungo (local name)*		–	–	–	–	0.56
25	*Maina (local name)*		–	–	–	–	1.08
26	*Terminalia alata*	Combretaceae	–	–	–	–	1.06

## Appendix I

Importance Value Index of Different species.
